# Pharmacological Interactions of Nintedanib and Pirfenidone in Patients with Idiopathic Pulmonary Fibrosis in Times of COVID-19 Pandemic

**DOI:** 10.3390/ph14080819

**Published:** 2021-08-20

**Authors:** José M. Serra López-Matencio, Manuel Gómez, Esther F. Vicente-Rabaneda, Miguel A. González-Gay, Julio Ancochea, Santos Castañeda

**Affiliations:** 1 Hospital Pharmacy Service, Princesa Hospital, IIS-Princesa, c/Diego de León 62, 28006 Madrid, Spain; josemaria.serra@salud.madrid.org; 2 Methodology Unit, Health Research Institute Princesa (IIS-IP), c/Diego de León 62, 28006 Madrid, Spain; mgomezgutierrez@salud.madrid.org; 3 Rheumatology Service, Princesa Hospital, IIS-Princesa, c/Diego de León 62, 28006 Madrid, Spain; efvicenter@gmail.com; 4 Rheumatology Service, Marqués de Valdecilla Universitary Hospital, University of Cantabria, Av. de Valdecilla 25, 39008 Santander, Spain; miguelaggay@hotmail.com; 5 Pneumology Service, Princesa Hospital, Autonomous University of Madrid (UAM), IIS-Princesa, c/Diego de León 62, 28006 Madrid, Spain; j.ancochea@separ.es; 6 Department of Medicine, Autonomous University of Madrid (UAM), 28029 Madrid, Spain

**Keywords:** antifibrotic agents, COVID-19, interstitial lung disease, IPF, progressive fibrosing ILD, UIP, pharmacological interactions

## Abstract

The discovery of antifibrotic agents have resulted in advances in the therapeutic management of idiopathic pulmonary fibrosis (IPF). Currently, nintedanib and pirfenidone have become the basis of IPF therapy based on the results of large randomized clinical trials showing their safety and efficacy in reducing disease advancement. However, the goal of completely halting disease progress has not been reached yet. Administering nintedanib with add-on pirfenidone is supposed to enhance the therapeutic benefit by simultaneously acting on two different pathogenic pathways. All this becomes more important in the context of the ongoing global pandemic of coronavirus disease 2019 (COVID-19) because of the fibrotic consequences following SARS-CoV-2 infection in some patients. However, little information is available about their drug–drug interaction, which is important mainly in polymedicated patients. The aim of this review is to describe the current management of progressive fibrosing interstitial lung diseases (PF-ILDs) in general and of IPF in particular, focusing on the pharmacokinetic drug-drug interactions between these two drugs and their relationship with other medications in patients with IPF.

## 1. Background

Interstitial lung diseases (ILD) are a heterogeneous group of pulmonary disorders characterized by varying degrees of inflammation and fibrosis resulting in the loss of alveolar function and impairment of gas exchange [1]. Idiopathic pulmonary fibrosis (IPF) is an entity that is included in the group of interstitial lung diseases of unknown etiology, being a severe form of pulmonary fibrosis that is associated with high morbidity and mortality [1,2,3].

IPF has a variable incidence depending on the population under study. Thus, in the United Kingdom, IPF has an incidence of around 7.44 cases per 100,000 inhabitants, while in the United States some series show an incidence of 16.3 cases per 100,000 inhabitants or even 93.7 cases per 100,000 people as described in a systematic review conducted by Hutchinson [4] covering the decade from 2001 to 2011. Overall, it is estimated that the worldwide prevalence may be close to 60 cases per 100,000 inhabitants. This entity predominantly affects males over 65 years of age [2,3,4].

Since its appearance, the coronavirus disease 2019 (COVID-19) pandemic has affected millions of people worldwide causing more than three million deaths. The available data indicate that a significant percentage of individuals suffering from severe acute respiratory syndrome caused by severe acute respiratory syndrome coronavirus-2 (SARS-CoV-2) develop acute lung injury/acute respiratory distress syndromes (ALI/ARDS), which can become severe. Pulmonary fibrosis is a recognized sequel of ARDS. Currently, there is evidence of fibrotic changes in radiographic images of patients recovered from COVID-19 [5,6,7].

Although IPF is the most widely studied and most common fibrosing ILD, there are also other progressive fibrosing (PF)-ILDs such as certain connective tissue disease-associated ILDs, which evolve towards pulmonary fibrosis and present a similar behavior to IPF, characterized by worsening of respiratory symptoms, decline in lung function and early mortality despite standard of care treatment [1,2,3]. In the same line, the PROGRESS study showed data on patients with other chronic PF-ILDs who were admitted to a hospital in Lyon, France, between 2010 and 2017. This study showed that those patients who had a loss of a quarter of their lung function or a loss of forced vital capacity (FVC) ≥ 10%, had 3 year survival rates of 83% and 5 year survival rates of 72%. In addition, some factors were shown to be associated with worse evolution, such as age > 70 years; FVC < 70% and/or diffusing capacity of the lungs for carbon monoxide (DLCO) < 40% at diagnosis; reduction in FVC ≥ 10% from the estimated value or decrease in DLCO ≥ 15% from the estimated value within 6–12 months of follow-up; and decrease > 50 m in the 6 min walking test at 6 months [8].

Indeed, much of the information given in this review is applicable to both IPF and other non-IPF ILDs with a fibrosing phenotype [1,2].

Regarding the pathophysiology of IPF and of other PF-ILDs, it is multifactorial and results in a progressive deterioration of lung function. Some risk factors for progression have been described, including environmental factors, microbial agents or some previous pathologies such as gastroesophageal reflux, which have a probable genetic basis that confers the patient a certain susceptibility to the disease [9].

IPF is the most common idiopathic interstitial pneumonia in the world. It is characterized by a heterogeneous, irreversible, progressive and unpredictable course associated with significant morbidity and poor prognosis after diagnosis [1,2,3]. There is growing evidence supporting that the disease originates from the interaction between the variable expression of genetic polymorphisms, changes related to cellular aging and exposure to certain environmental factors, such as smoking, industrial powders, chronic gastric microaspiration, viral infections and possibly alterations in the lung microbiome [1,3]. The lesions produced by repetitive exposures aberrantly activate the alveolar epithelial cells of genetically susceptible individuals, promoting apoptosis of the epithelium, recruitment of mesenchymal cells and increased vascular permeability. Unregulated epithelial/mesenchymal interaction results in the secretion of a variety of profibrotic cytokines, metalloproteinases and procoagulant mediators, which promote uncontrolled migration and proliferation, and differentiation in fibroblasts to myofibroblasts as well as fibrosis in the extracellular matrix. The main pro-inflammatory cytokines involved in fibrosis are tumour necrosis factor (TNF)-α and interleukin (IL)-1, as well as some fibrous factors such as transforming growth factor (TGF)-β and platelet-derived growth factor (PDGF) [1,2,3,4,5].

Patients present a nonspecific symptomatology, which is the fundamental cause of the delay in diagnosis. Accordingly, in order to reach a definitive diagnosis, it is essential to combine a detailed medical history with the realization of radiological imaging studies and sometimes with histopathological studies obtained through a pulmonary biopsy (PB). Currently, the gold standard for diagnosis of IPF and other non-IPF PF-ILDs is multidisciplinary discussions that can improve the precision of diagnosis, avoiding unnecessary tests such as pulmonary biopsy and optimizing patient management. The multidisciplinary team should include a pulmonologist, a radiologist, a pathologist, a thoracic surgeon, a rheumatologist and a specialist nurse [10].

Patients with IPF present with dyspnea, cough and asthenia, which are symptoms that cause a reduction in daily physical activity and muscle strength leading to a precarious quality of life and often result in social isolation with increased levels of dependence and immobility as the disease progresses and causing a significant number of cardiopulmonary complications. In addition, these patients experience depressive and anxiety disorders, creating a situation that is difficult to manage for both patients and their caregivers [11].

Another cause for the delay in the diagnosis of IPF is that this is an entity that can be easily confused with other respiratory pathologies requiring multidisciplinary assessment by the pulmonology, radiology and pathological anatomy services, thereby using more healthcare resources [10,12].

Lung transplantation is the only therapeutic option that appears to increase the life expectancy of patients with IPF. This procedure would be indicated when there is a higher probability of accelerated decrease in FVC and, therefore, a poor prognosis in the short term. As the knowledge of IPF has deepened and several technical advances have been achieved, especially in the area of transplant immunology and surgical procedures (involving both means and technique), the average age of recipients undergoing lung transplantation has increased in recent decades from 45 to 55 years. However, this therapeutic option has been extended in recent years to patients up to 65 years of age in specialized centers. Nevertheless, despite these advances, pulmonary recipients with IPF have an overall survival rate upon single transplantation between 4 and 5.5 years, depending on the series, and may exceed 10 years for bilateral pulmonary transplantation [10,11,12].

Traditionally, IPF treatment was based on immunosuppressants, glucocorticoids, oxygen therapy and palliative measures. However, the PANTHER-IPF study showed that treatment with azathioprine, N-acetyl cysteine and prednisone was associated with increased hospitalizations and mortality [13]. Currently, there are two drugs approved for this pathology that have been shown to delay lung deterioration associated with the disease with satisfactory safety and tolerability profiles. These two drugs are nintedanib and pirfenidone [14,15]. However, there is little information on the pharmacological interactions of these two agents in IPF patients who are usually polymedicated.

The group of COVID-19 patients most affected by severe disease present clinical characteristics highly similar to patients suffering from PF-ILD, rendering PF-ILD management more important than ever [5,6,7].

Below we review the main pharmacological interactions of the two currently available antifibrotic drugs, used individually or in combination, as well as some practical aspects of their therapeutic management, which have become more complex in this pandemic.

## 2. COVID-19 and ILD

SARS-CoV (Severe Acute Respiratory Syndrome Coronavirus) and MERS-CoV (Middle East Respiratory Syndrome Coronavirus) are genetically similar to SARS-CoV-2 and cause lung syndromes similar to COVID-19. At the end of the SARS pandemic on June 2003, 8422 people were affected and 916 died. On the other hand, MERS, which began in April 2012, infected 2519 recognized subjects out of which 866 died. Tomographic abnormalities in SARS included the following: rapidly progressive ground-glass opacities, some of them with consolidation of some regions of the lung; and apparent reticular changes approximately two weeks after the onset of symptoms, which persisted in half of the patients for about 4 weeks. A 15 year follow-up study of 71 patients with SARS showed that interstitial and functional abnormalities progressively decreased, resulting in recovery after the first 2 years following infection and then remained stable; at 15 years, only one patient had obstructive pulmonary disease and none had restrictive respiratory dysfunction, while 4–6% showed interstitial abnormalities [6]. Similar to the findings in SARS, ILD with a fibrosing phenotype has been reported in MERS [7].

Several cases of patients with severe pneumonia of unknown cause appeared in Hubei province, China, in December 2019. Almost one month later, these cases were reported to the World Health Organization (WHO). They started an outbreak that was later declared a pandemic by WHO. The causative agent of this disease was identified as a betacoronavirus RNA, similar to SARS-CoV, which was thus called SARS-CoV-2. This coronavirus causes lung, gastrointestinal and neurological disease. It has a diameter between 60 and 140 nanometers and is covered by an envelope formed by different spicules, which gives it a solar corona appearance. By genetic recombination, coronaviruses acquire the capacity to infect any host, including bats and humans. SARS-CoV-2 is able to infect the nasal and bronchial epithelia, as well as pneumocytes, through the binding of the spike (S) protein of viral spicules to its receptor on the cell surface, which is the angiotensin-converting enzyme-2 (ACE-2); this interaction triggers an inflammatory response and, subsequently, the clinical picture of pneumonia and/or respiratory failure [1,6]. In one of the first studies conducted in China during the pandemic, the characteristics of 1099 patients were reported. Out of these, 173 cases were severe, with an average age of 49 years; 57% were male; 28% were smokers; and 23.7% (over 70 years) presented comorbidities such as diabetes, hypertension and chronic obstructive pulmonary disease (COPD); 5% of the cases were in intensive care; 2.3% on mechanical ventilation; and 1.4% of patients died [16]. As of June 2021, 180,569,000 infections and 3,912,200 deaths have been reported worldwide [17].

Fibrotic changes have been found in chest computerized tomography (CT) in patients with COVID-19. Available data indicate that one-third of the recovered patients develop fibrotic abnormalities, 47% have impaired DLCO and 25% have decreased total lung capacity. In a study by Huang et al., all patients who survived had varying degrees of fibrotic damage ranging from subtle linear opacities to diffuse distribution of crazy paving pattern, with extensive fibrosis evidenced in 52% of patients. In another study by Zhou et al. including 62 patients, 21 (33.9%) had fibrotic changes, which were more likely to occur in advanced stages of the disease (8 to 14 days from onset of symptoms) than in earlier stages (less than 7 days). Similarly, Pan et al. reported fibrotic changes in chest CT in 11 out of 63 patients during acute illness. These reshapings are supported by autopsy reports. Early fibrotic changes in the course of the disease suggest repair attempts following lung damage; all this results in pulmonary sequelae, which include impaired exercise capacity, fibrotic lung tissue and impaired diffusing capacity. However, although pulmonary function can be improved over time, moderate fibrosis could be irreversible in some patients [18,19]. Thus, it would make sense to apply the same strategy as in other non-IPF PF-ILDs in these patients and antifibrotic agents could play a relevant role.

The early identification of subpopulations of patients developing PF-ILD phenotype after COVID-19 infection is important since it is presumed that, by acting early in the course of ARDS, the development of lung damage could be avoided, delayed or decreased [18]. Several markers associated with mortality risk including age, disease severity, time of Intensive Care Unit (ICU) stay, mechanical ventilation and hyperinflammatory markers may be potential predictors of PF-ILD. Other factors such as male sex, smoking and underlying diseases have also been described. In addition, prolonged fever prior to hospital admission, tachypnea and eosinopenia at admission may be useful as a combination of early risk indicators [19].

Age: Pulmonary fibrosis is most often reported in elderly individuals. The exact reason for this association is unknown; however, older individuals are more susceptible to SARS, MERS and SARS-CoV-2 and are more likely to possess severe symptoms [17].

Disease severity: According to the WHO, 80% of COVID-19 cases are mild, 14% develop severe symptoms and 6% are very severe. Comorbidities such as high blood pressure, diabetes and coronary artery disease are factors associated with increased severity. Laboratory findings that correlate with increased severity are as follows: lymphocytopenia, leukocytosis and lactate dehydrogenase (LDH) increase. Serum LDH levels have been used as a marker of disease severity following acute lung damage. LDH is an indicator of lung tissue destruction and correlates with mortality risk. Peak LDH levels were significantly correlated with the risk of pulmonary fibrosis after infection in MERS and SARS [17]. In a meta-analysis, Chen et al. reported that elevated LDH values were associated with a 12-fold increase in the risk for severe COVID-19 and concluded that LDH levels can be used to predict severe disease [20].

Time of hospitalization at the ICU and mechanical ventilation: Five percent to twelve percent of COVID-19 patients required ICU admission. Although disease severity is closely related to the time of hospitalization at the ICU, mechanical ventilation provides an additional risk of ventilator-induced lung damage. Ventilator-associated lung damage is an acute damage that is initiated or exacerbated by mechanical ventilation and is associated with increased mortality in ARDS. Pressure and volume abnormalities induce this damage, resulting in the release of proinflammatory modulators, worsening of acute lung damage, increased mortality and pulmonary fibrosis in survivors. In a follow-up study of 27 patients with ARDS who received mechanical ventilation, 23 (87%) had pulmonary fibrosis between 110 and 267 days after extubation [17].

Smoking: It is associated with chronic oxidative stress, increased expression of inflammatory cytokines and pulmonary fibrosis. The harm associated with smoking continues even after smoking cessation. A systematic review by Vardavas and Nikitara showed that smokers are 1.4 times more likely to have more severe symptoms of COVID-19 and 2.4 times more likely to need the ICU, mechanical ventilation or die than non-smokers [17].

Chronic Alcoholism: Alcohol abuse is associated with recurrent pneumonia due to the aspiration of gastric contents. Clinical and experimental studies show that alcoholism causes glutathione depletion, chronic oxidative stress, inflammation and induction of TGF-B in the lungs, thereby increasing the risk of acute lung injury and pulmonary fibrosis [17].

Patients should be advised not to leave the house and to use non-face-to-face methods for consultations (telemedicine), to obtain medicine stocks (they can be formulated for 3 months) and, if required, to ask for help in order to avoid leaving the house (from family or friends). They should also take into account the different recommendations on fever, odynophagia, dry cough and dyspnoea for 1 week and consult for suspected COVID-19. A management strategy should be established with patients and family members, if possible, with recommendations on how to proceed during a mild exacerbation at home, including indications about warning signs for them to attend emergencies or to contact their physician and reminding them that they may not necessarily be infected with COVID-19. Medications for interstitial lung disease should be maintained at the dose recommended by the attending physician but should be discontinued at the time of acute COVID-19 infection in order to avoid drug interaction or side effects. The patient’s immune response appears to play an important role in the pathophysiology of both acute lung injury and ARDS. Patients with COVID-19, particularly those with pneumonia and ARDS have elevated levels of proinflammatory cytokines and other inflammatory biomarkers. Currently, the most commonly used drugs for the acute phase of COVID-19 are glucocorticoids. In fact, in the RECOVERY trial, dexamethasone has shown a moderate but significant reduction in mortality among those patients who were receiving either invasive mechanical ventilation or oxygen alone at randomization but not among those receiving no respiratory support. However, despite this clinical trial being one of the most robust studies regarding the use of glucocorticoids in COVID-19, its methodology is somehow questionable among other reasons because no severity markers were recorded. Furthermore, several routes of administration of dexamethasone, oral or intravenous, were used [21]. Notwithstanding the need of further evidence, glucocorticoids seem to be the cornerstone of the treatment of the acute phase of COVID-19 to date. Additionally, the combination of supportive therapy along with antiviral treatment, oxygen therapy and anticoagulation must be emphasized [22]. In order to clarify the role of anti-inflammatory and immunomodulatory treatment of the acute phases of COVID-19 on the occurrence of long-COVID and post-ARDS interstitial lung disease, further research is needed. Finally, pulmonary rehabilitation in the acute and inflammatory phases is essential for the full recovery of lung function in these patients.

## 3. Pharmacovigilance

According to the WHO, pharmacovigilance is defined as “the science and activities related to the detection, assessment, understanding and prevention of adverse drug effects or any other possible drug-related problem”. The safety system covers adverse drug reactions (ADR) produced by medications, dosing errors, falsified medicinal products, their lack of effectiveness and misuse and/or abuse and drug interactions, among others. It also involves monitoring the safety of natural and traditional medicines, blood products, radioactive substances, contrast media, biological products, vaccines and even medical devices [23]. The main purpose of pharmacovigilance is to determine the cause, frequency and severity of ADRs in such a manner that the necessary preventive measures can be put in place in order to preserve patient safety and achieve the rational use of medicinal products optimizing the benefit/risk ratio. Therefore, it is considered a key piece for ensuring the efficiency and effectiveness of the pharmaceutical regulatory systems, clinical practice and the programmes implemented in healthcare [24].

### Drug Interactions

When a medicine is administered, it undergoes a series of processes that contribute in inducing its therapeutics and toxic effects in the organism and are summarized under the acronym LADME (liberation, absorption, distribution, metabolism and excretion). A drug interaction occurs when the concomitant administration of two drugs alters any of the aforementioned processes [25]. There are several scenarios resulting from a drug–drug interaction: drug absorption can be delayed, decreased or increased; the distribution within the body and the pharmacological effect for which the drugs were designed may be altered; or their metabolism and/or excretion can be significantly modified [26].

The understanding of the mechanism involved in a given drug interaction is essential for its interpretation, prevention and treatment. However, it is not easy to establish a clear mechanism for each interaction since they usually involve more than one drug acting simultaneously through different mechanisms [27]. Two main groups of drug interactions can be considered:

1. Pharmacodynamic: They take place at biologically active sites, such as receptors, and produce changes in pharmacological activity. They do not usually affect pharmacokinetic parameters, but they alter the patient’s response to the drug. These interactions are as clinically important as pharmacokinetic interactions but much more difficult to study systematically since they usually take place affecting pairs of medications, which makes it difficult to establish common mechanisms explaining the effects on both drugs. Two types of pharmacodynamic interactions can be defined [26]:
  - Synergistic: Two drugs with the same pharmacological effect are administered together;
  - Antagonistic: Two drugs that are administered together have opposite actions.
2. Pharmacokinetic: They affect different drug kinetic processes, resulting in modifications in plasma drug concentration. There are different types of pharmacokinetic interactions depending on whether they occur at the level of absorption, distribution, metabolism or excretion [26,28].

Absorption: They can affect both the speed and magnitude of absorption. In general, these interactions have little clinical relevance and can be avoided by separating the administration of the two drugs.

Distribution: Displacement of plasma protein binding. They occur when two drugs compete for the same binding site in plasma proteins; in this case, the drug with the lowest affinity for the protein is displaced by the one with the highest affinity. The result is an increase in the concentration of free (active) drug, which is usually compensated by an increase in its excretion. These interactions are only clinically important for drugs in which the percentage of plasma protein binding is greater than 90%.

Metabolism: The interactions at this level are the most important from a clinical point of view. Cytochrome P-450 is the main responsible for the metabolism of drugs, as well as other exogenous substances (polycyclic aromatic hydrocarbons, etc.) and endogenous compounds (steroids, hormones, prostaglandins, lipids and fatty acids), through mono-oxidation reactions.

The term cytochrome P-450 refers to a group of numerous isoenzymes located in the membrane of the smooth endoplasmic reticulum of hepatocytes. They are also present at high concentrations in small intestine enterocytes and in small amounts in extra-hepatic tissues, such as kidney, lung and brain. Cytochrome P-450 enzymes form a genetic superfamily that can be divided into families and subfamilies. To date, more than 30 different isoenzymes have been identified in humans, but 90% of oxidation reactions can be attributed to the six main families: CYP1A2, CYP2C9, CYP2C19, CYP2D6, CYP2E1 and CYP3A4.

Interactions affecting the metabolism are caused by the induction or inhibition of cytochrome P-450 isoenzymes. Enzymatic induction is a gradual process since it requires the synthesis of new enzymes and produces a decrease in the plasma level of the drug being metabolized. Enzymatic inhibition, on the other hand, takes place more quickly and results in an increase in the plasma concentration of the affected drug. A drug can be metabolized simultaneously by more than one isoenzyme. In addition, a drug does not need to be a substrate of this enzyme to behave as an inducer or inhibitor of a specific isoenzyme.

Excretion: Those drugs that alter the renal excretion of other drugs can affect their plasma levels. The two most common mechanisms of interaction at the renal level are competition for active tubular secretion and the modification of urinary pH. The clinical impact of these types of interactions depends on the percentage of renal elimination of a drug or its metabolites, but these mechanisms are not as important as those involving the metabolism in general.

It should be emphasized that the probability of drug interactions increases substantially with the number drugs administered simultaneously to a patient. Accordingly, drug interactions are expected to be greater in the polymedicated patient. Therefore, drug interactions go hand-in hand with polymedication [29,30]. The WHO defines chronic diseases as “diseases of long duration and usually of slow progression” [31]. Heart disease, stroke, respiratory diseases, cancer and diabetes are good examples of chronic diseases. Between 60% and 70% of deaths worldwide are attributed to these diseases. In turn, 80% of these deaths occur in low-income and middle-income countries inhabited by a large part of the global population, affecting important aspects of the lives of both men and women. Other examples of chronic diseases include hearing and visual impairments, oral diseases, osteoarticular diseases, gene and mental disorders. The main risk factors for the development of chronic disease are smoking, an unhealthy diet, physical inactivity and alcoholism [31].

## 4. Pirfenidone

Pirfenidone (5 methyl-1-phenyl-2-[1H] pyridone) is an agent that combines anti-inflammatory and antifibrotic effects acting on the regulation of TGF-5 activity, TNF-α and β pathways, as well as cellular oxidation. Pirfenidone is indicated for the treatment of mild to moderate IPF [15]. Pirfenidone was approved for IPF based mainly on the two CAPACITY trials [32] (Table 1). Moreover, in order to confirm the beneficial effect of pirfenidone on disease progression, another trial was performed showing positive outcomes [33] (Table 1). According to the RELIEF study, inpatients with fibrotic ILDs other than IPF (such as connective tissue disease-associated ILDs, fibrotic non-specific interstitial pneumonia, chronic hypersensitivity pneumonitis and pneumoconiosis) attended in 17 centers specialized in ILD in Germany deteriorated despite conventional therapy. However, with the addition of pirfenidone to the existing treatment there was an attenuation of disease progression, as measured by decline in FVC [34]. Nevertheless, more studies for assessing the efficacy and safety of pirfenidone in this kind of patients are needed.

Pharmacologically, pirfenidone belongs to the group of immunosuppressive agents. It was approved by the US Food and Drug Administration (FDA) and the European Medicines Agency (EMA) in 2011, becoming the first drug authorized for the treatment of IPF. Pirfenidone has antifibrotic and anti-inflammatory properties both in vitro and in animal models of pulmonary fibrosis [15]. In addition, pirfenidone reduces the accumulation of inflammatory cells and attenuates the proliferation of fibroblasts, the production of cytokines and proteins related to fibrosis and the increased synthesis and accumulation of extracellular matrix [15].

Treatment of mild to moderate IPF with pirfenidone in adults begins with 800 mg/day and is increased up to 2400 mg/day over the course of 3 weeks. The most common adverse reactions are observed at the gastrointestinal, skin and liver level (Table 2). It is worth highlighting that polymedicated patients should be closely monitored, as pirfenidone metabolism can be influenced in these patients by inhibition or induction of liver enzyme systems such as cytochrome P450 1A2 (CYP1A2), CYP3A4 and P-glycoprotein (P-gp) [15]. Table 2 shows a list of drugs that should be avoided when initiating pirfenidone treatment because the risk/gravity of their adverse effects outweighs the benefit from treatment (major interactions).

Various analyses of cumulative information from several studies have provided several important conclusions [40,41]. For instance, in a single-dose drug interaction study involving 27 volunteers, co-administration of 800 mg pirfenidone and 750 mg ciprofloxacin (moderate CYP1A2 inhibitor) two times daily for 6 days increased exposure to pirfenidone by 81% [15] (Table 1). Consequently, in the case of strong CYP1A2 inhibitors such as fluvoxamine or enoxacin, the dose of pirfenidone should be reduced to one-third of the usual dose, while for moderate inhibitors such as ciprofloxacin, it should be reduced to 66% of the commonly used dose [15].

For P450 1A2 inducers, a study in which a single pirfenidone dose of 801 mg was administered to 25 healthy non-smoking patients and 25 smokers without concomitant therapy at the time of the study showed less exposure to the drug in smoking subjects (area under the curve (AUC) 46% in smokers and 68% in non-smokers) (Table 1) [40].

Pirfenidone has been shown to exert low inhibition (10% to 30%) of P-gp-mediated digoxin (5.0 μM) efflux at concentrations of 100 M and higher [8]. The inhibitory activity of pirfenidone for CYP2C9 and 2C19 or 1A2, 2D6 and 3A4 was evaluated in vitro at a concentration of 1000 μM (approximately 10 times the average of the maximum concentration (Cmax) in humans). The activity of these enzymes was reduced by 30.4%, 27.5%, 34.1%, 21% and 9.6%, respectively; this effect has great clinical relevance, as it increases Cmax and AUC values proportionally to the dose used [41].

## 5. Nintedanib

Nintedanib is an intracellular tyrosine kinase inhibitor developed for the treatment of various types of cancer (lung, ovary, renal, colo-rectal and liver), as well as an antifibrotic agent. The first clinical trial that confirmed the effects of nintedanib slowing the progression of idiopathic pulmonary fibrosis was the phase II TOMORROW [35], which was confirmed in the phase III INPULSIS-1 and INPULSIS-2 [36].

In 2014, it was approved for the treatment of IPF in the USA and Europe, and it received a new indication for systemic sclerosis (SSc)-associated ILD (SSc-ILD) therapy in 2019 [37]. More recently, this drug has been approved for the treatment of other progressive fibrosing interstitial lung diseases (PF-ILD) [38].

Nintedanib is a potent oral inhibitor of the tyrosine-kinase activity of several pro-angiogenic receptors: vascular endothelial growth factor receptors (VEGFR) 1–3, fibroblast growth factor receptors (FGFR) 1–3 and platelet-derived growth factor receptors (PDGFR) α and β. Additionally, it inhibits the kinase activity of RET receptors, FLT3 and the Src family of tyrosine kinases. Overall, more than 12 tyrosine-kinase receptors and signalling molecules are inhibited by nintedanib, suggesting potential effects on multiple signalling pathways [14].

Nintedanib displays linear pharmacokinetics for a dose of 350 mg twice daily. The maximum plasma concentrations are normally reached 2–4 h after oral administration with food. Its half-life is 9.5 h (Table 2), and the average concentration in the steady state is normally reached in one week, with low concentrations remaining stable for a period longer than 1 year. The absolute bioavailability of nintedanib 100 mg is 4.7%. This pharmacokinetic profile is due to the quick metabolization of the molecule by methylesterases [14,35,36].

The main metabolic pathway of nintedanib is esterase-mediated hydrolysis followed by glucuronidation. CYP450, primarily CYP3A4, plays a minor role in nintedanib biotransformation. The main route of nintedanib elimination is the bile/faecal pathway (which accounts for about 93% of the administered dose). The contribution of renal excretion to total clearance is low at about 0.65% [14]. The co-administration of nintedanib with ketoconazole, a CYP3A4 and P-gp inhibitor, increases nintedanib exposure by 60%; patients receiving nintedanib concomitantly with P-gp and CYP3A4 inhibitors should be closely monitored. The co-administration of nintedanib and rifampicin, a P-gp and CYP3A4 inducer, decreases nintedanib exposure by 50%. Therefore, concomitant administration of nintedanib with P-gp or CYP3A4 inducers should be avoided [14,35,36]. Table 2 shows a list of drugs that should be taken into account when treating patients with nintedanib due to the risk of major drug interactions.

## 6. Managing the Adverse Effects of Antifibrotic Therapy

The most common adverse events (AEs) of antifibrotic therapy occur at the gastrointestinal tract [14,15] (Table 2). Grouped data from TOMORROW and INPULSIS trials (Table 1) showed that the AE most commonly associated with daily nintedanib 300 mg was diarrhoea, reported in 61.5% of cases (17.9% with placebo) [13,42]. In most patients, nintedanib-associated gastrointestinal AEs can be managed by reducing the dose (200 mg/day), discontinuing treatment and applying symptomatic measures with loperamide or similar [14]. The most common AE associated with pirfenidone in the CAPACITY and ASCEND studies was nausea, which appeared in 35.5% of patients vs. 15.1% in patients treated with placebo [43]. Gastrointestinal toxicity associated with pirfenidone is managed by reducing the dose or interrupting treatment [14]. Taking treatment after meals can also be helpful [43]. Photosensitivity and rash associated with pirfenidone appear mostly in the first months of treatment. In CAPACITY and ASCEND trials, rash was reported in 29.2% of patients treated with pirfenidone compared to 9% in patients treated with placebo [43]. This AE can be reduced by the use of photoprotective creams [14]. Table 3 shows recommended strategies for the prevention and treatment of the most common AEs associated with antifibrotic agents.

In addition, nintedanib and pirfenidone may cause an increase in liver enzymes. Therefore, it is necessary to perform control analyses at the start of treatment as well as periodically during treatment for the early detection of potential liver damage [14,15,44,45]. Dose adjustments made to manage AEs do not reduce the effectiveness of nintedanib or pirfenidone decreasing FVC [46,47,48,49].

Results from a study of 186 patients from a single centre in the United States showed that the percentage of patients who had to discontinue treatment with pirfenidone or nintedanib due to AEs was similar to that observed in clinical trials (20.9% and 26.3%, respectively), with gastrointestinal AEs being primarily responsible for discontinuation [49]. Moreover, several studies have shown that efficacy and safety data in clinical practice are similar to those described in clinical trials [50,51,52,53,54].

## 7. Concomitant Administration of Nintedanib and Pirfenidone

AUC and Cmax values obtained for nintedanib administration in conjunction with pirfenidone in IPF patients, indicate that there are no clinically relevant interactions between these two agents. Although exposure to nintedanib in these studies decreased when administered with pirfenidone compared to monotherapy, this fact lacks clinical relevance and can be attributed to the great inter-individual variability among patients, as it is also observed when nintedanib is administered as monotherapy [55,56].

Regarding pharmacokinetics of pirfenidone, the AUC and Cmax values are similar when this drug is used alone or in combination with nintedanib [55,56]. This information was more consistent in the INJOURNEY study, which evaluated the safety, efficacy and pharmacokinetic profile of nintedanib alone or in combination with pirfenidone. Interestingly, combined treatment reduced FVC less than nintedanib in monotherapy, although these data should be interpreted with caution [39].

As both drugs produce similar AEs [4,5], a negative additive effect could be expected when they are administered together. However, the AEs of the combined administration were similar to those of individual treatments [57,58].

The data analysed until now suggest the absence of relevant clinical interactions between these two drugs. Although there is a lot of information regarding long-term individual administration of pirfenidone and nintedanib [57,58], information about their combined administration is very limited. There is only a small Phase IV study in 20 patients with IPF under long-term treatment with both drugs (Table 1), in which no added AEs were observed [39].

Cost-effectiveness remains to be improved, as the association of two antifibrotic agents considerably increases the cost of treatment per patient and year, thereby increasing the economic burden on healthcare providers. In recent years, inhaled administration of new antifibrotic agents has been explored; this new administration route could improve adherence to treatment in polymedicated patients [59].

## 8. Final Considerations

In December 2019, reports emerged from Wuhan, China, of a new severe acute respiratory disease caused by SARS-CoV-2. COVID-19 pneumonia presents as an acute respiratory infection, with fever, dry cough, dyspnoea, arthralgia and other symptoms, which may be similar to some interstitial lung diseases. Therefore, history, epidemiological link, physical examination and clinical examination of the patient should be taken into account for a correct differential diagnosis. In this manner, when a physician faces a patient with this type of interstitial lung disease, it is important to define the complete medical history in order to determine whether the interstitial disease is acute or chronic and to consider the diagnosis of infection by COVID-19, which is, perhaps, the greatest challenge [5,6,7].

Data from previous coronavirus-induced diseases such as SARS and MERS, as well as emerging data from the COVID-19 pandemic, suggest there could be substantial fibrotic consequences following SARS-CoV-2 infection. Nintedanib and pirfenidone might have a role in preventing severe lung fibrosis after SARS-CoV-2 infection, especially in patients with the PF-ILD phenotype [5,6,7,60]. Furthermore, we must take into account that these drugs do not produce immunosuppression, which is an advantage over the use of corticosteroids in the acute phase of the disease. This becomes particularly important in patients at high risk of contracting any type of infection during COVID treatment. Thus, switching to antifibrotic therapy is an option to be considered in some patients.

In addition, chronic diseases are a growing threat capable of triggering serious repercussions ranging from negatively affecting health-related quality of life and being an underestimated cause of poverty for both families and society in general to being the leading cause of premature deaths worldwide [60]. It is important to note that these population groups are especially susceptible to interactions, mainly because of polypharmacy. Polymedication is a frequent phenomenon in chronic diseases and, in the majority of cases, is associated with non-compliance with treatment, inappropriate use and/or abuse of drugs, dosing mistakes or inadequate medication, among others. Its prevalence is an imminent alarm in the health sector as it has undergone significant growth in the last years [60].

IPF and PF-ILDs are chronic diseases affecting patients who often take at least four different kinds of drugs [61]. In these patients, the risk of a harmful and unwanted response increases exponentially, resulting in new requirements for dealing with conditions wrongly interpreted as “new pathological processes”, which give rise to a therapeutic cascade. Moreover, drug interactions are more frequent in the polymedicated patient [62].

Although not all drug interactions are clinically significant, it is important to remain vigilant for those that are relevant. It is impossible to keep in mind all the relevant interactions described, but knowledge of the main types of drugs most frequently involved in interactions can be crucial for establishing an alert system that contributes to improving prescription of drug therapy. Several methods that allow the reduction in the risks associated with interactions and thus improve the therapeutic risk/benefit ratio have been described, including among others the reduction in the number of drugs administered, avoiding unnecessary polymedication, the selection of alternative drugs with low AE rate, an adequate dosing regimen adjusted to the individualized characteristics of every patient, pharmacokinetic monitoring of serum concentrations of drugs in those cases in which this is possible and constant clinical observations in order to detect the consequences of an interaction as quickly as possible [62,63,64,65].

Nintedanib and pirfenidone have represented a breakthrough in the treatment of IPF in recent years. Their different mechanisms of action open the possibility of using them in combination, thus providing an interesting therapeutic option, especially for those cases with worse prognosis. However, little information is currently available on the possible pharmacological interactions that could occur in the case of combined administration and on the additive effects that this interaction could exert on the effectiveness and safety of both drugs.

## Figures and Tables

**Table 1:** Main clinical trials on pirfenidone and nintedanib in idiopathic pulmonary fibrosis (IPF).

Study (References)	Design	Treatment	Main Endpoints	Patients
CAPACITY 004 [32]	Phase 3 Randomized Parallel Assignment Double-Blind	Pirfenidone (2403 mg or 1197 mg) versus Placebo	Absolute Change in Percentage of predicted FVC Mean Change in Percent Predicted FVC as measured from baseline to week 72	435
CAPACITY 006 [32]	Phase 3 Randomized Parallel Assigment Double-Blind	Pirfenidone (2403 mg) versus Placebo	Change in percentage of predicted FVC at week 72	344
ASCEND [33]	Phase 3 Randomized Parallel Assigment Double-Blind	Pirfenidone (2403 mg) versus Placebo	Change in FVC or death at week 52	555
RELIEF [34]	Phase 2 Randomized Parallel assignment Double blinded	Pirfenidone (267 mg or 534 mg or 801 mg) versus placebo	Absolute change in percentage of predicted FVC at week 48	127
TOMORROW [35]	Phase 2 Randomized Parallel assignment Double blinded	Nintedanib (50 mg,100 mg, 200 mg or 300 mg) versus Placebo	Annual rate of decline in FVC over 52 weeks	432
INPULSIS 1- INPULSIS 2 [36]	Phase 3 Randomized Parallel assignment Double blinded	Nintedanib (200 mg or 300 mg) versus Placebo	Annual rate of decline in FVC over 52 weeks	1066
SENSCIS [37]	Phase 3 Randomized Parallel assigment Double blinded	Nintedanib (150 mg) versus placebo	Annual rate of decline in FVC over 52 weeks	576
INBUILD [38]	Phase 3 Randomized Parallel assigment Double blinded	Nintedanib (150 mg) versus placebo	Annual rate of decline in FVC over 52 weeks	663
INJOURNEY [39]	Phase 4 Randomized Parallel assignment Open-label	Nintedanib (150 mg) versus Pirfenidone (2403 mg)	Percentage of patients with on-treatment gastrointestinal AEs from baseline to week 12	105

Abbreviations: FVC: forced vital capacity. AE: Adverse Events.

**Table 2:** Pharmacological characteristics of nintedanib and pirfenidone.

	Pirfenidone	Nintedanib
Pharmaceutical form (orally)	Capsules Tablets	Capsules
Half-life (hours)	3	9.5
Side effects	Bloating, dizziness, diarrhoea, dyspepsia, gastroesophageal reflux, nausea, vomiting, fatigue, weight loss, photosensitivity reactions and rash	Increased liver enzymes, abdominal pain, diarrhoea, nausea, vomiting weight loss
Major pharmacological Interactions *	Aminolevulinic acid, amiodarone, enoxacin, fluvoxamine, leflunomide, mibefradil, mipomersen, rucaparib, teriflunomide, vemurafenib	Carbamazepine, dexamethasone, drotrecogin alfa, phenytoin, leflunomide, lomitapide, mipomersen, mitotane, phenobarbital, primidone, rifampicin, St. John’s wort, tripanavir, teriflunomide
Contraindications	Smoking Kidney failure Liver failure	Thromboembolic disease Lung toxicity Gastric perforation Smoking Kidney failure Liver failure
Pregnancy Category (FDA)	C	D

Abbreviations: Pregnancy category C: Animal reproduction studies have shown adverse effects on the foetus or its safety could not be demonstrated. There are no adequate and well-controlled studies in humans. Drugs included in this category should only be used when the potential benefits justify the potential risks to the foetus. Pregnancy category D: There is evidence of risk to the foetus based on research data, post-marketing data, adverse reaction records or human studies. However, the potential benefits of its use in pregnant women may be acceptable despite the likely risks in some situations. FDA: Food and Drug Administration. * Major interactions: Highly relevant at the clinical level. Combinations causing this type of interactions should be avoided since their risk exceeds the potential benefit.

**Table 3:** Strategy for the prevention and management of adverse events related to antifibrotic therapy.

	Pirfenidone			Nintedanib	
**Type of AE**	**Gastrointestinal**	**Cutaneous**	**Hepatic**	**Gastrointestinal**	**Hepatic**
AE prevention	Take pirfenidone with plenty of food. Titration for 4 weeks instead of 2.	Avoid exposure to sunlight or intense artificial light. Applications of complete protection cream every 2 h. Use of sunglasses and protective clothing. Avoid use of phototoxic drugs.	Monitor liver bio-chemistry (ALT, AST and bilirubin) at baseline, monthly for 6 months and then every 3 months.	Take nintedanib with food.	Monitor liver biochemistry (ALT, AST, bilirubin) at baseline, monthly for the first 3 months and then periodically.
AE treatment	Prokinetics and proton pump inhibitors.	Steroids or sulphadiazine if severe phototoxicity.		Antidiarrheal (loperamide). Antiemetics. Proper hydration.	
Dose reduction	Reduce doses to 1–2 capsules 2–3 times daily. Make the reduction at the time point in which the AE is most pronounced.	Reduce dose to 1 capsule every 8 h for one week.	If AST and ALT are increased (>3 to 5× ULN) or there are symptoms or hyperbilirubinemia, reduce doses until values recovery.	Reduce to 100 mg/12 h if persistent diarrhoea.	If AST/ALT are increased (>3 to 5× ULN) reduce dosage until values recovery. Then, re-scale doses up to max tolerated.
Dose interruption	If AE persists, temporarily discontinue therapy until symptom resolution.	Discontinue doses for 14 days if rash persists and subsequently re-escalate. Do not re-escalate if the rash does not subside.	Permanently discontinue if the elevations of AST and ALT are accompanied by symptoms of hyperbilirubinemia or if the elevations are >5× ULN.	Stop doses if severe diarrhoea for one week. Discontinue permanently if there is no improvement.	Permanently stop doses if elevations are accompanied by severe symptoms of liver damage.

Abbreviations: AE: adverse events; ALT: alanine amino-transferase; AST: aspartate amino transferase; max.: maximum; ULN: upper limit of normality.

## Data Availability

Data sharing not applicable.
